# Femtosecond laser-assisted selective reduction of neovascularization in rat cornea

**DOI:** 10.1007/s10103-014-1545-0

**Published:** 2014-02-26

**Authors:** Mehra S. Sidhu, Min-Yeong Choi, Suk-Yi Woo, Hyun-Kyu Lee, Heung-Soon Lee, Kyu-Jin Kim, Sae Chae Jeoung, Jun-Sub Choi, Choun-Ki Joo, Il-Hong Park

**Affiliations:** 1 Center for Medical Metrology, Division of Convergence Technology, Korea Research Institute of Standards and Science, Daejeon, 305 340 Republic of Korea; 2 Department of Medical Physics, University of Science and Technology, Daejeon, 305 350 Republic of Korea; 3 Department of Ophthalmology and Visual Sciences, Seoul St. Mary’s Hospital, The Catholic University of Korea and The Catholic Institute for Visual Science, Seoul, 137 701 Republic of Korea; 4 HanbitNanoBioTech. Co., Ltd., Seoul, 152 848 Republic of Korea

**Keywords:** Rat, Cornea, Corneal neovascularization, Femtosecond laser, Microsurgery

## Abstract

Nonlinear multiphoton absorption induced by focusing near infrared (NIR) femtosecond (fs) laser pulses into a transparent cornea allows surgery on neovascular structures with minimal collateral damage. In this report, we introduce an fs laser-based microsurgery for selective treatment of rat corneal neovascularizations (in vivo). Contiguous tissue effects are achieved by scanning a focused laser pulse below the corneal surface with a fluence range of 2.2–8.6 J/cm^2^. The minimal visible laser lesion (MVL) threshold determined over the corneal neovascular structures was found to be 4.3 J/cm^2^. Histological and optical coherence tomography examinations of the anterior segment after laser irradiations show localized degeneration of neovascular structures without any unexpected change in adjacent tissues. Furthermore, an approximately 30 % reduction in corneal neovascularizations was observed after 5 days of fs laser exposure. The femtosecond laser is thus a promising tool for minimally invasive intrastromal surgery with the aid of a significantly smaller and more deterministic photodisruptive energy threshold for the interaction between the fs laser pulse and corneal neovascular structures.

## Introduction

Corneal neovascularization (CNV) is a common and serious complication of many corneal diseases including inflammatory disorders, infectious keratitis, contact lens-related hypoxia, alkali burns, stromal ulceration, and limbal stem cell deficiency. Within the intrastromal region, angiogenesis is a complex multistage process where the sprouting of new blood vessels takes place from the pre-existing vasculature [1, 2]. This is further associated with severe visual impairment and represents a major public health problem [3]. The primary treatment for actively proliferating corneal vessels is topical corticosteroids. However, in corneas where vessels have been established for an extended period, corticosteroid treatment is known to be less effective [4]. Numerous laser treatment methods such as photocoagulation and verteporfin or indocyanin green (ICG)-mediated photodynamic therapy (PDT) have also been devised for the removal of CNVs. However, severe adverse effects have been frequently reported for the current treatments due to a high incidence of recanalization and thermal damage to adjacent tissues [4–9].

Femtosecond (fs) laser ablation of a material meanwhile yields negligible thermal damage [10, 11]. The damage induced by the fs laser is furthermore restricted to the size of the laser spot with a localized ablation depth (*l*), which is estimated by the expression *l* = *δ* ln (*F* / *F*_th_^*δ*^), where *F*_th_^*δ*^ and *δ* are the ablation threshold fluence and optical penetration depth, respectively [10, 12, 13]. Furthermore, the photochemical effect induced by free electrons generated through multiphoton absorption dominates at a lower fluence range (<25.3 J/cm^2^ for porcine retina) [12]. However, at a higher range of fluence, apparent thermomechanical disruptive effects on the targeted material appear [11]. A low ablation threshold upon irradiating a material with an ultrafast laser pulse is thus generally favorable for minimizing possible photo-induced damage near the ablation area [14, 15]. In addition, local ablation of various materials such as biological tissues or metals can be efficiently achieved if the laser fluence is precisely controlled [6, 12, 14–17]. With systematic control of the fs laser fluence, induction of either transient or permanent changes in various cellular compartments including cell walls, plasma membranes, and even organelles is possible. Note also that the incident beam can be tightly focused by using a high numerical aperture objective at a subfemtoliter focal volume [18–21]. Ultrafast laser microsurgery has recently been utilized in selective removal of angiogenesis in early developmental stages of zebrafish embryos [22]. The major clinical application of near infrared (NIR) fs lasers includes blade-free corneal flap creation prior to refractive surgery [23]. The effects of different parameters including the pulse energy [13, 16], laser repetition rate [24], wavelength [15], pulse width [14, 17], and tissue depth [14] on the operation performance have been widely investigated. fs-pulsed lasers are also employed in other clinical procedures such as lenticule formation [25], keratoplasty [26], lentotomy [27], intra-tissue refractive index shaping [28], and trabecular meshwork surgery [29]. However, to our knowledge, their utilization in treatment of angiogenesis without the involvement of any photodynamic or chemical agents in the anterior segment of the eyes has not been explored [4].

In this report, we introduce the usage of the fs laser as a microsurgical tool for specific treatment on the neovascular structures in Norway brown rat corneas (*Rattus norvegicus*) under in vivo conditions, where no agents were applied. The system used in the current study is equipped with devices for ophthalmoscope-assisted real-time monitoring of microsurgery along with acquisition of images while irradiating the target [19]. Corneal neovascularization was artificially induced within the intrastromal region of the rat cornea to imitate the disease environment using the silver nitrate cauterization model. The fs laser-assisted microsurgery system enables us to selectively expose the neovascular structures formed on the corneal stroma of the rat eye. After the laser operation, the disruption of corneal neovascular structures was closely examined with the help of optical coherence tomography (OCT) and a postoperative histological analysis. Finally, we observed the animals for 5 days after the fs laser operation in order to record short-term changes and the reduction of neovascular structures within the corneal stroma.

## Material and methods

### Animals

Thirty-two adult (200–250 g) brown Norway rats (*R. norvegicus*) (Orient Bio Inc., South Korea) were used to evaluate the effects of fs laser exposure. Mature Norway brown rats were maintained under standard laboratory conditions (12 h light–12 h dark cycle). The average heart rate of the rats was 300 to 400 bpm, with a respiratory rate of about 100 cpm [30]. It takes about 600 ms for one respiratory cycle, causing a single movement in the rat body that only pertains to its breathing activity. The movements were quantified by an analysis of real-time videos recorded at a rate of 30 fps before, during, and after exposure of fs laser irradiation. After determining the correlation statistics, the fs-pulsed irradiations were synchronized so as to maintain uniformity of the exposure.

All the fs treatments were performed with strict adherence to the “Guidelines for Animal Care and Experimentation” prepared by the Korean Animal Protection Law (2008) [31] and the Catholic Hospital, Seoul (Korea) Animal Care Committee. For all procedures, animals received the injectable anesthetics Zoletil 50 and Rompun (Virbac Laboratories, France) with a ratio of 3:1, given at a dose of 1 ml/kg of body wt., intraperitoneally. Before the experiments, a few drops of topical anesthetic Alcaine (proparacaine HCl 0.5 %) were applied to halt eye muscle movements. To prevent the corneal tissue from drying, ringer solution was topically applied at regular time intervals. Maintenance amounts (10–15 %) of anesthetics were administered at 45 min intervals, if necessary. Phenylephrine 2.5 % was topically administered to stimulate the pupillary dilation.

### Induction of corneal neovascularization

**Fig. 1:**
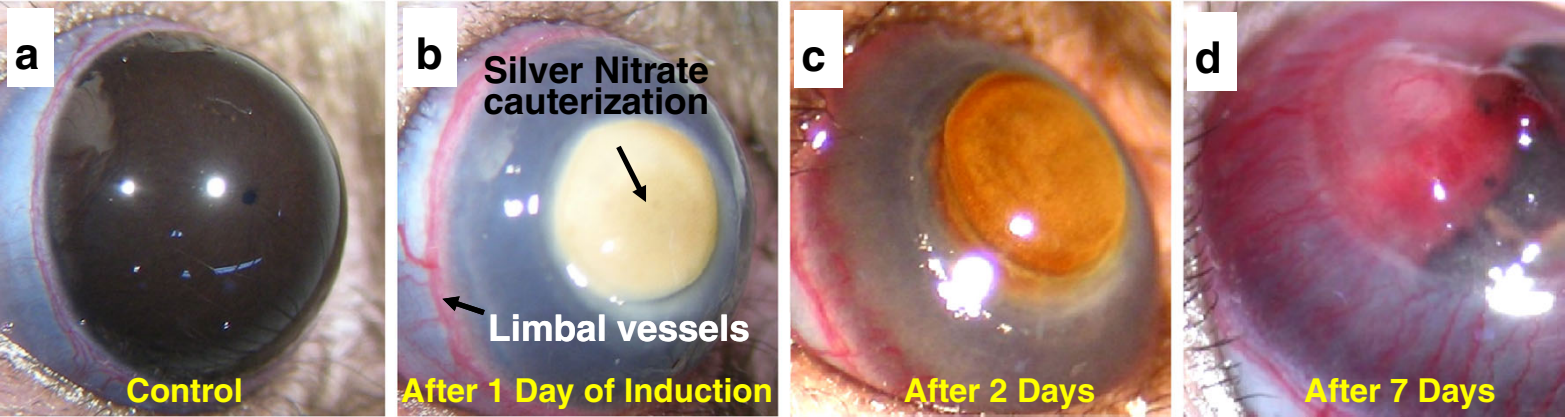
Optical images of rat cornea before (**a**) and after silver nitrate cauterization (**b**–**d**) to induce corneal neovascularization

For induction of corneal neovascularization (CNV) in the rat eye, the silver nitrate cauterization model was employed. Both eyes of each test animal were cauterized by pressing an applicator stick coated with 75 % silver nitrate and 25 % potassium nitrate (Sigma Aldrich, USA) to the center of the cornea for about 5 s [2, 32, 33]. Following the cauterization, a discrete area of damage to the epithelial and stromal cell layers at the corneal surface was seen as a yellowish brown patch, in contrast with control rat corneas having no induction of angiogenesis (Fig. 1a, b). After 24 h of cauterization, the corneas became increasingly thickened while surrounding limbal vessels also appeared edematic, as shown in Fig. 1b [2]. Within 48 h, numerous short vascular sprouts were projecting from the limbal vessels toward the site of injury (Fig. 1c). By the 3rd day of intervention, thick dense neovascular structures had elongated evenly into the cornea from all sides of the cornea. After 7 days of induction (Fig. 1d), regression and remodeling of these new blood vessels was observed, whereas few stable and mature vessels could be sustained longer, even after 10 days of intervention. As a whole, the model recalls all the major steps followed in blood vessel formation in a reproducible manner. Therefore, it could be successfully utilized to imitate the diseased environment. Rats were divided into two subgroups. Here, 16 subjects were selected for fs laser-assisted microsurgery treatment after 2 days of induction and an equivalent number (16) were kept as a control. All the neovascular structures subjected to the fs laser treatment were in the early stage of development. Anterior segment photography was performed under anesthetic conditions using a high-resolution digital camera to document the pre- and postoperative changes in rat corneas.

### Femtosecond microsurgery system

**Fig. 2:**
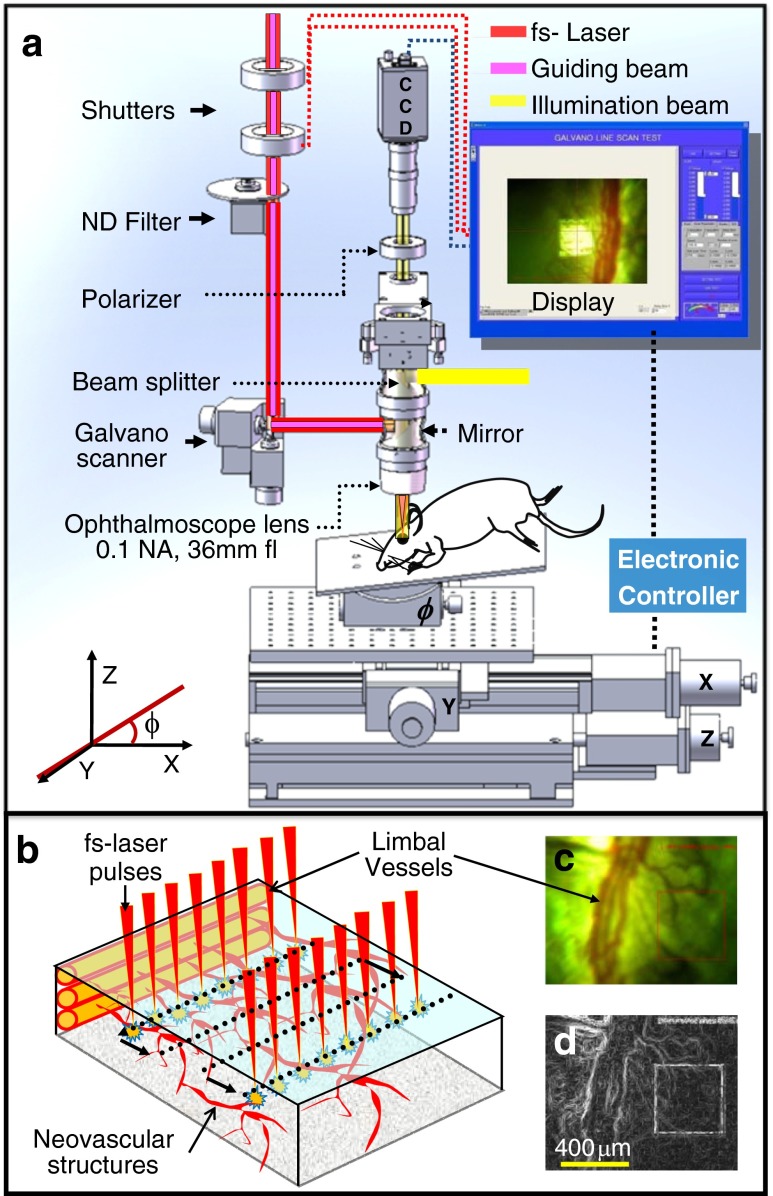
**a** Schematic diagram of fs laser-assisted rat corneal angiogenesis treatment system. XYZ translation stage and tilt mounting were used to align the rat eyes to the fs laser beam direction. **b** Schematic representation of scanning pattern of fs laser pulses into the corneal stroma. **c** Optical image of rat cornea captured before exposure of fs laser irradiation. **d** Transformed image of rat cornea using the Prewitt mask operator to distinguish the neovascular structures. *Scale bar* is 400 μm

Figure 2 presents a schematic diagram for fs laser-assisted microsurgery system for the treatment of neovascular structures within the rat corneal stroma. The fundamental output from a regenerative amplified Ti-sapphire (*λ* = 810 nm) laser with a pulse width of 150 fs and a repetition rate of 1 kHz (Libra, Coherent Inc., USA) was focused into the corneal stroma region of the rat eye by using an ophthalmoscope lens with a focal length of 36 mm (Carl Zeiss Inc., Germany). The numerical aperture (N.A) was 0.16. The plane of focus was matched with the plane of the neovascular structures in reference to the peripheral limbal vessels. No applanation optics was applied while focusing the fs laser irradiation into the corneal stroma. The laser spot was almost circular with a diameter of 7.6 μm (*measured at 1/e* ^*2*^ *in intensity*). The system includes a software-controlled laser aiming system with an XY-Galvano scanner to track pre-assigned targets visualized in optical images of the rat cornea. A fast-switching light emitting diode (LED) (CREE XR-E Q5, 180 lm) was used as the illumination source to obtain image sequences with a rate of 30 fps [19]. A guiding beam (695 nm, Melles Griot Inc. USA) along with a surgical (fs) and illumination beam (LED) was passed inline through the ophthalmoscope lens, allowing us to map the focal position at the plane of neovascularization.

The rat body, under anesthetic conditions, was placed on a motorized XYZ translation stage, used to manipulate the target to expose a fresh area of tissue at each laser scan. The stage was tilted at an angle (*ϕ*) ranging from 45° to 60° to align neovascular structures and limbal vessels at a right angle to the fs laser direction. The *X*–*Y* plane of limbal vessels and CNVs were matched to the plane of lab coordinates, as shown in Fig. 2b. This allows us to precisely focus the laser beam onto neovascular structures. Figure 2b also presents the scanning pattern and depth, which is kept at the focal plane of neovascular structures. Panels c and d of Fig. 2 show optical and corresponding transformed images of the rat cornea before fs laser exposure, respectively. The optical image was transformed by using a Prewitt mask operator, making it possible to distinguish the edges of neovascular structures at the focal plane [19].

Meanwhile, to estimate the minimal visible laser (MVL) lesion threshold for corneal neovascularizations, the laser fluence was varied from 2.2 to 8.6 J/cm^2^. The area of a scan was 150 × 150 μm and the number of incident laser pulses (about 400) was kept constant. However, for reduction of neovascular structures over a larger region, the scan area was increased 7-fold (400 × 400 μm). Correspondingly, the laser shot numbers were also increased seven times (~2,704) so that the laser shot density would be constant for all experiments. All experiments were conducted under a single scan configuration, which provides a single shot environment on the surface without any overlap to avoid any multiple shots, considering both the laser beam diameter at a focal point of about 7.6 μm and the number of laser pulses per unit scan length. The rat corneas of control samples (*without any angiogenic induction*) were also exposed to verify the side effects of fs laser irradiation. After fs laser irradiation, the samples were monitored for 3 min to check postoperational bleeding effects from abnormal blood vessels.

Optical images of anterior segments were taken at 1, 2, and 5 days after the laser exposure and analyzed by using the freely available software ImageJ 1.46, available from http://imagej.nih.gov/ijand developed by Wayen Rasband, National Institute of Mental Health, USA. The percent red intensity (Red%) for each treated, untreated, and control sample was defined by the following expression:

1 $$ \mathrm{Red}\%=\frac{R}{\left(R+G+B\right) / 3}\times 100 $$

where *R*, *G*, and *B* represent red, green, and blue intensities of the selected area [5]. We defined the % reduction of neovascularization as follows:

2 $$ \%\mathrm{reduction}=\frac{\mathrm{Red}\%\left(\mathrm{untreated}\right)-\mathrm{Red}\%\left(\mathrm{treated}\right)}{\mathrm{Red}\%\left(\mathrm{untreated}\right)}\times 100 $$

where Red%(untreated) and Red%(treated) are the percent red color intensity estimated from the positive control rat cornea and fs laser-exposed cornea, respectively.

### Optical coherence tomography

Under anesthetic conditions, before and after exposure of the fs laser on corneal neovascularization, OCT images of rat corneas were recorded by using SR-OCT (Spectral Radar OCT, ThorLabs, USA). The refractive index was set to 1.4, which is nearly equivalent to water, due to the relative composition of the cornea [5]. The axial resolution for the measurement is 4.5 μm. Sequential sections were recorded with an interval of 50 μm from the center of the cornea up to the surrounding limbal vessels for the control as well as the samples containing neovascular structures.

### Histological studies and SEM imaging

Histological analyses were conducted for laser-treated rat corneas. After exposure, the animals were euthanized using asphyxiation in a CO_2_ chamber for subsequent histological analysis of lesions induced by fs laser pulse irradiation. Rat eyes were enucleated, cleaned, and fixed in 4 % *p*-formaldehyde overnight. The samples were embedded in a tissue freezing medium (paraffin wax) for 24 h [34]. The tissues were rapidly frozen further at −20 °C. Afterwards, serial cross corneal sections (*t* = 6 μm) were cut using a cryostat (CM1850, Leica Microsystems, Germany). Sections were placed on microscope slides (SuperFrost -20; Matsunami Glass Inc., Japan) and air-dried. The sections were stained with hematoxylin and eosin using the reported protocols [12, 16–19]. Histopathologic findings from optical microscopic examinations were recorded (Zeiss Axioscope 2). In order to resolve the disruption of the endothelial lining of abnormal blood vessels, the stained cryosections were further coated with a thin layer of Pt to measure the scanning electron microscopic images. Lower electron energy (<1 kV) was employed to avoid any possible tissue damage.

## Results and discussion

### Removal of corneal neovascular structures

**Fig. 3:**
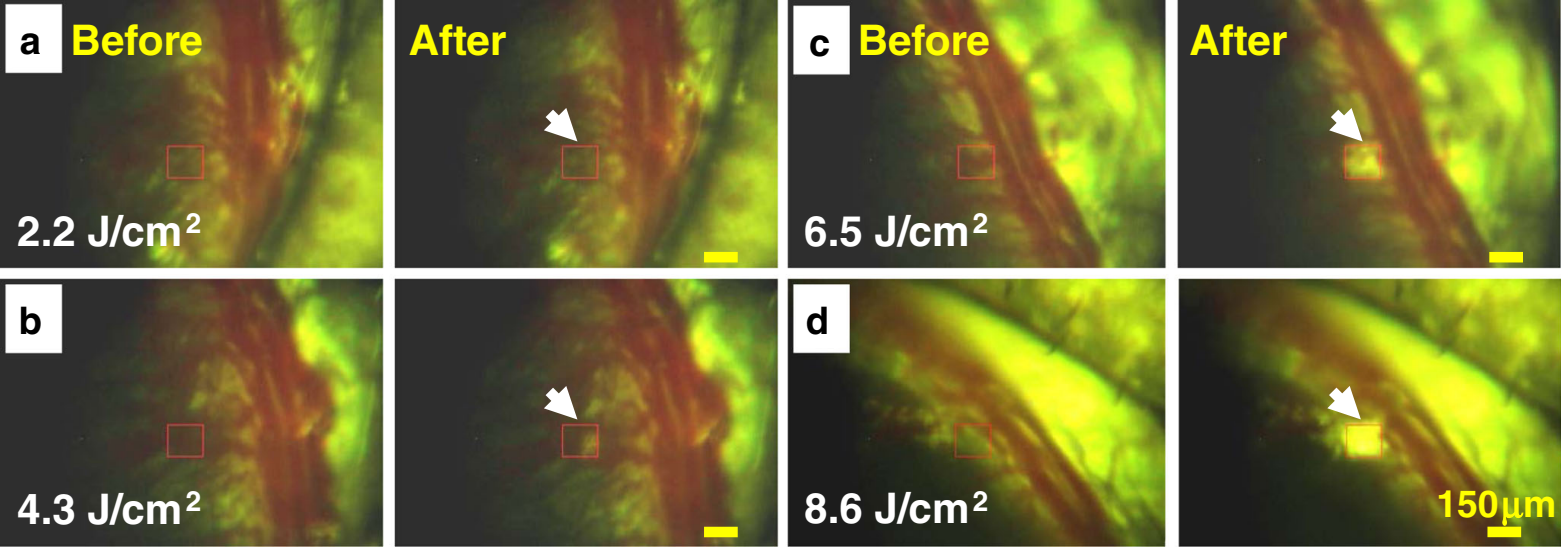
Sequential optical images taken from Norway brown rat cornea before and after the exposure of fs laser irradiation with variation of the laser fluence of 2.2 J/cm^2^ (**a**), 4.3 J/cm^2^ (**b**), 6.5 J/cm^2^ (**c**), and 8.6 J/cm^2^ (**d**). The scan area indicated by *red-colored squares* is 150 × 150 μm. *Scale bar* is 150 μm

While obtaining corneal images from Norway brown rat eye at the optimized focal plane and the polarizer angle with the aid of sharpness algorithm [19] at a rate of about 30 fps, we produced a series of laser lesions into the cornea containing neovascular structures. Initially, the following scanning parameters were employed to determine the MVL lesion threshold over the CNV with a scanning area of 150 × 150 μm and total scanning time of ~426 ms. The total number of equally distributed (in the *X*- and *Y*-axes) laser shots employed was ~400, i.e., the interval between consecutive laser shots was about 7.5 μm, which is close to the laser beam diameter. The MVL lesion ablation threshold over the neovascular structure was determined from high-resolution CCD images captured before and after the fs laser exposure (Fig. 3). The laser fluence was varied from 2.2 to 8.6 J/cm^2^. In order to verify the MVL threshold, four to five replications at each fluence were made. For each scan, the tissue was displaced and refocused with the aid of the XYZ translation stage prior to the laser exposure. Any noticeable changes observed on neovascular structures in comparison to the adjacent intrastromal region immediately after the laser exposure were categorized as an indication of damage. The first visible detectable lesion was found at a fluence of 4.3 J/cm^2^ (Fig. 3b) and is referred to as the minimal visible laser lesion threshold. There was no observable damage found on either the intrastromal region or the neovascular structures at fluences less than 2.2 J/cm^2^. When the set laser fluence was greater than 6.5 J/cm^2^, the size of the lesions increased such that it covered the entire scanning area. For currently used femtosecond exposures, the laser pulse duration is shorter than the electron cooling and recombination times [11]. Thus, the minimal energy is nonlinearly absorbed during the pulse into the focused portion of tissues. However, the time scale of absorption is much shorter than both the thermal diffusion and shockwave propagation times. This might lead to localized photodisruption effects and subsequent reduction of stromal damage within the vicinity of laser focus [5, 6, 11]. fs ultrashort pulsed lasers for enclosure of corneal neovascularization in the presence of ICG at 3.8 J/cm^2^ was employed by Sawa et al. in 2004 [35]. The MVL threshold values determined in the current study are in good agreement with previous reports [12, 19, 35], despite that no dye or photodynamic chemical agents were applied during the procedures.

In another in vivo experiment, the choriocapillaris of normal rabbits was closed by using a continuous wave (cw) diode laser tuned to 810 nm at fluence as low as 6.3 J/cm^2^ with the assistance of ICG at rates of 10 and 20 mg/kg [36]. However, after 9 days of exposure, inner photoreceptor segments showed degeneration in addition to the disruption of retinal pigment epithelium cells. This region also exhibited a loss of photoreceptor external segments. Rather, long pulse duration of millisecond range and large spot size (0.8–4.0 mm) resulted in collateral damage within the target tissues. Meanwhile, Sun et al. [14] reported a square root relationship between thresholds of laser-induced optical breakdown (LIOB) in porcine corneas and the laser pulse widths, under an in vitro environment. The LIOB threshold in porcine cornea was 4.8 J/cm^2^ for 20 ps pulse width and declined to 1.16 and 0.6 J/cm^2^ for 1.6 ps and 800 fs pulse widths, respectively [14]. The LIOB for ps pulses from a ND:YAG laser (1,064 nm, 10 Hz) in bovine cornea, lens, and vitreous was also about 12-fold lower than that for nanosecond (ns) pulses generated from the same laser system [17].

**Fig. 4:**
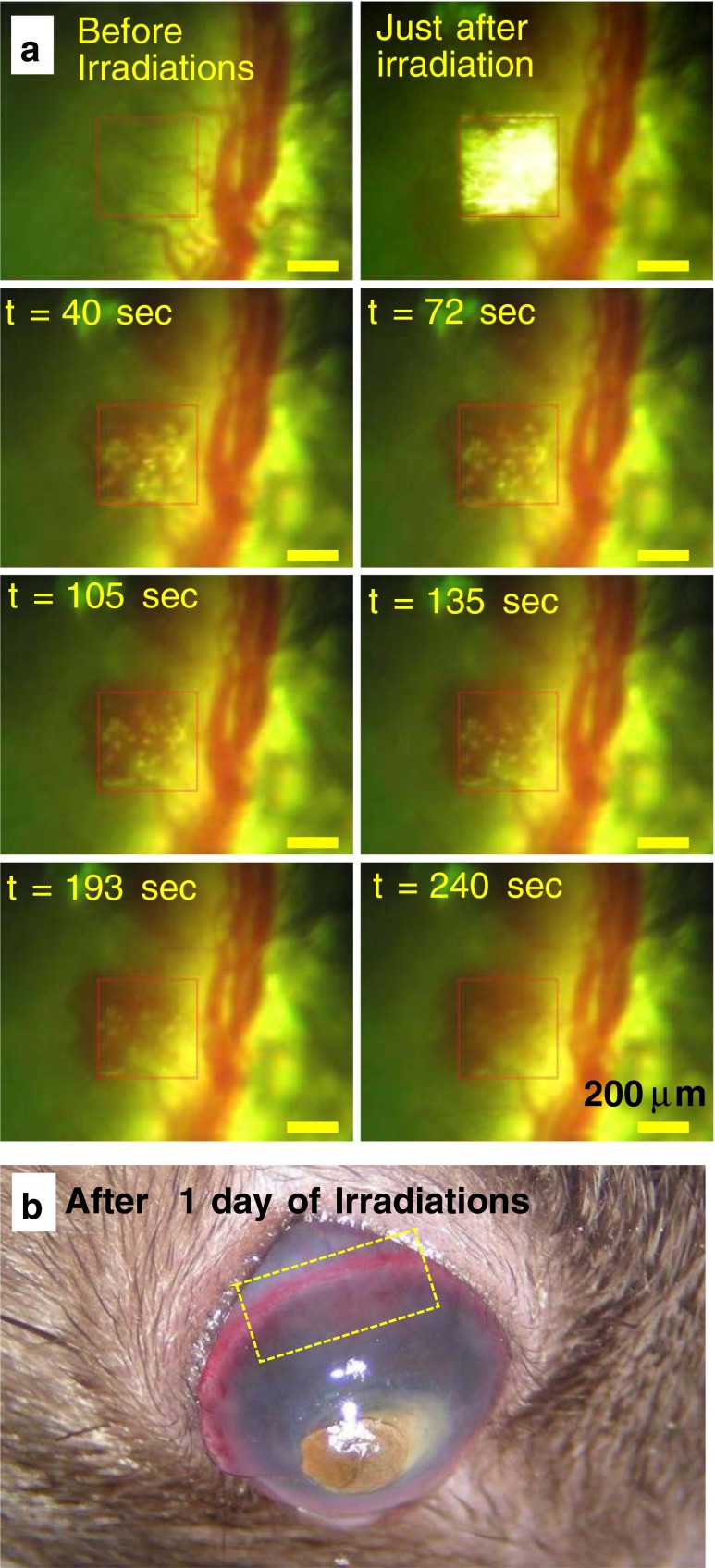
**a** Sequentially taken optical images of blood flow from a corneal blood vessel after exposure to single scan fs laser pulse irradiation with fluence of 8.6 J/cm^2^. The elapsed time after laser irradiation is denoted in each image. The laser pulses are focused at the area indicated by *red-colored squares* (400 × 400 μm). The *scale bar* is 200 μm. Optical image of rat eye after 1 day (**b**) of fs laser exposure. The area of treatment is marked by a *yellow box*

To study the blood outflow statistics, high laser fluence greater than 6.5 J/cm^2^ was employed. The area of exposure (400 × 400 μm) was increased 7-fold rather than the original exposure area of 150 × 150 μm. Since the shot density is kept constant, the successive laser spots should not be overlapped. After a single exposure of fs laser irradiation, apparent disruption of neovascular structures followed by continuous outflow of blood within tens of seconds was observed, as shown in Fig. 4a. The outflow of blood from the vascular rupture completely stopped within 3 min after the laser irradiation. However, at lower laser fluence, no bleeding was observed. After a day of fs laser exposure, the bleeding was stopped (Fig. 4b). There is no further continued hemorrhage from the site of laser treatment. The outflowed blood or debris from neovascular structures could be removed by regenerating corneal cells, which have a much greater phagocytosis activity compared to unstimulated corneal cells [37].

**Fig. 5:**
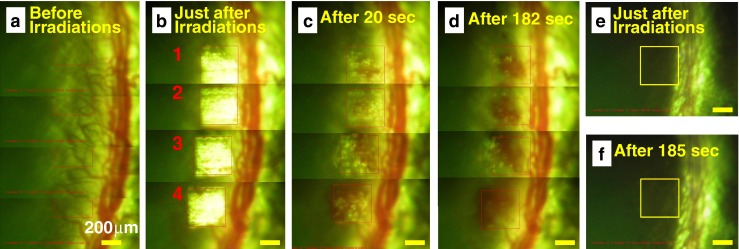
Sequential optical images of corneal neovascular structures induced by silver nitrate cauterizations model. The images were serially captured before (**a**), just after (**b**), and time elapsed after (**c**, **d**) the exposure of fs laser pulses. The capture time is denoted in the lower panel for each image. The laser fluence at the site of treatment is 8.6 J/cm^2^. CCD captured images for the control rat cornea just after (**e**) and time elapsed after (f) the exposure of fs laser irradiation. *Scale bar* is 200 μm

In agreement with the current work, the vascular damage without any distortion within rat brain parenchyma (in vivo) as well as primate trabecular meshwork (ex vivo) showed the dependence of laser-induced breakdown on the laser fluence applied during microsurgery treatments [18–21, 29]. At the lowest energies, extravasation was detected without any severe bleeding. However, with increasing laser energy, vessel rupture was induced, resulting in hemorrhages and the outflow of blood.

fs laser pulses were further exposed to larger target area to reduce the neovascular structures over a wider corneal region. Rats were selected for the fs laser treatment 2 days after silver nitrate cauterization on the cornea. Optical images were captured to mark the region of interest within the rat cornea (Fig. 5a). The parameters including scanning area of 400 × 400 μm, fluence of 8.6 J/cm^2^, and number of laser spots of 52 × 52 for treatment were the same as discussed in the previous section. Four successive scans were carried out to cover larger surface area (Fig. 5b). Before each laser irradiation, the surgical laser (fs) beam was focused over the neovascular structures by guiding laser-assisted focusing. The motorized XYZ translation stage was used to manipulate the rat cornea position, allowing exposure of a fresh area of corneal tissue for each laser scan. After the treatment, the site of each scan was monitored for ~3 min to observe the post-treatment changes to the corneal neovascular structures (Fig. 5c, d). As discussed previously, outflow of blood was observed immediately after the laser exposure. However, no further bleeding was observed after 3 min. There was no significant change or distortion observed on either the rat corneal surface or stroma except the disruption of neovascular structures, even though the fluence applied is two times higher than the MVL threshold. Further, fs lasers have a high potential to be utilized as a microsurgical tool. In a similar manner, the control samples (*rat corneas without induction of angiogenesis*) were also treated and monitored for 3 min after the exposure (Fig. 5e, f). No adverse effects of fs pulse irradiation were observed within the corneal stroma having no vascular structures. All the samples were further characterized by OCT and histological techniques to closely examine the effects of fs pulse laser irradiation.

### Postoperative characterizations of rat cornea

**Fig. 6:**
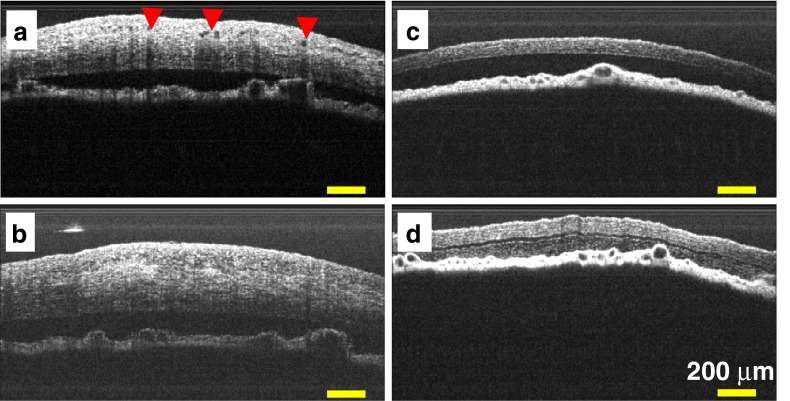
**a**, **b** Scanned OCT images of rat cornea, of which neovascularization induced by silver nitrate cauterization, before and after exposure of fs laser irradiation, respectively. **c**, **d** OCT images of control rat cornea before and after the exposure of fs laser irradiation, respectively. The corneal neovascularizations are indicated by *red arrow heads. Scale bar* is 200 μm

After the exposure of fs laser irradiation into the corneal stroma containing neovascular structures, we have sequentially imaged the rat corneas using optical coherence tomography. It has been reported that OCT, used to render tissue planes with high axial resolution, has potential for evaluating the cornea [38]. The CNVs were clearly observed as voids or empty spaces within the intrastromal region, having an order of tens of micrometers (indicated by red arrow heads in Fig. 6a). As shown in Fig. 6b after exposure of fs pulse irradiations, a significant reduction of voids or empty spaces was observed within the corneal stroma. Meanwhile, the images taken from the control rat show no presence of vascular structures (Fig. 6c). With the fs laser irradiation of the corneal stroma of control rat eyes, as shown in Fig. 6d), the thickness of the treated cornea was increased, possibly due to a wound healing response of the rat body [32, 34]. There is also a possibility of some hemorrhaging, which could cause some blurriness-like effects in the aqueous humor cavity of the rat cornea; however, no other substantial damage was observed in the current OCT images. These hemorrhages disappeared 1 day after the laser exposure due to phagocytic activity within the anterior segment of the rat eye [37]. It should be noted that the effects of fs laser irradiation were limited to the shallow region of corneal stroma since we have focused the fs laser pulses into rather turbid corneal stroma over the CNVs emerging from surrounding limbal vessels. No optical filamentation, which usually happened in transparent media when ultrafast laser is focused, is observed under the current fs laser surgical procedure [39, 40]. The optical coherence tomography images (Fig. 6a, b) show no such effect in the underlying corneal stroma layer and iris region.

**Fig. 7:**
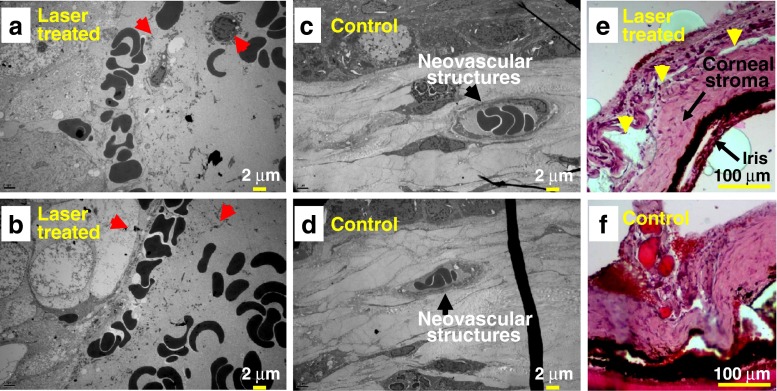
Scanning electron micrographs (SEM) of thin histological sections with a thickness of 6 μm of rat cornea collected 1 day after fs laser irradiation with laser fluence of 8.6 J/cm^2^. **a**, **b** The fs laser-irradiated rat eye shows apparent vascular hemorrhage as well as disruption of neovascular structures (*red arrows*), while **c**, **d** the control rat eye shows intact neovascular structures (*black arrows*). *Scale bar* is 2 μm. Optical micrographs of H & E-stained cryosections of rat cornea with fs laser irradiation (*yellow arrows*) (**e**) and the control (**f**) are also shown. *Scale bar* is 100 μm

A histological evaluation of rat cornea was carried out to verify the reduction of neovascular structures within the intrastromal region. Treated as well as untreated rats were sacrificed 1 day after fs laser exposure. Figure 7 shows scanning electron micrographs (SEM) and photomicrographs of the sectioned rat cornea with a thickness of 6 μm. The SEM images in Fig. 7a, b and the optical image in Fig. 7e taken after fs laser irradiation show the disruption of the endothelial lining of the neovascular structures and eventually the appearance of many extracellular blood cells in the corneal intrastromal region. Except the presence of red blood cells in the corneal stroma and destruction of neovascular structures, the epithelium, endothelium, iris, and corneal stroma were normal in appearance. This could be referred as selectivity. Meanwhile, the SEM micrographs of sectioned untreated rat corneas in Fig. 7c, d and the optical image in Fig. 7f reveal the presence of vascular structures.

The results from the current histological and OCT analyses suggest that fs laser irradiation offers selectivity for the photodisruption of neovascular structures within the rat cornea. The selectivity may come from the difference in damage threshold for the different nature of targeted tissues [12, 17–19, 22, 29]. We have already reported that the quaternary blood vessels with wall thickness of about 6 μm of enucleated porcine retina have optoperforation threshold fluence about four times lower than that for retina itself. We supposed that rather strong linear absorption of NIR fs laser pulse due to the presence of red blood cells in neovasculature also plays a role in their perforations [22]. These findings are in good agreement with in vivo studies performed in zebrafish embryos and the microsurgeries of various cellular compartments including cell walls, plasma membranes, and even organelles [15, 18, 20–22, 25, 41, 42]. Tsen et al. [43] also reported that if wavelength and pulse width of the excitation fs lasers were appropriately selected, there exists a window in power density that enables one to achieve selective inactivation of target viruses and bacteria without causing any apparent cytotoxicity in mammalian cells.

Ablations induced by fs pulse lasers can be generally grouped into two regimes: (1) strong ablations dominated by thermal vaporizations at intensities significantly higher than the threshold and (2) gentle ablations governed by the Coulomb explosions near the ablation threshold associated with either microcavitation or chemical ionization [44, 45]. The latter case is applicable to the current work. Previous in vivo studies on zebrafish embryos further suggest that the nature of vasculature also plays a vital role in the actual photo-induced disruptions of vascular structures [22]. The wall thickness of vascular structures at early stages of development is less than that at latter stages. Thus, at the early stage of neovascularization, the vascular structures were strongly affected by fs pulse irradiations [14, 22, 41]. The mechanical properties of biological tissues such as elasticity and mechanical strength of tissues could also modulate the kinetics and dynamics of ablation processes [11].

### Reduction of corneal neovascular structures

**Fig. 8:**
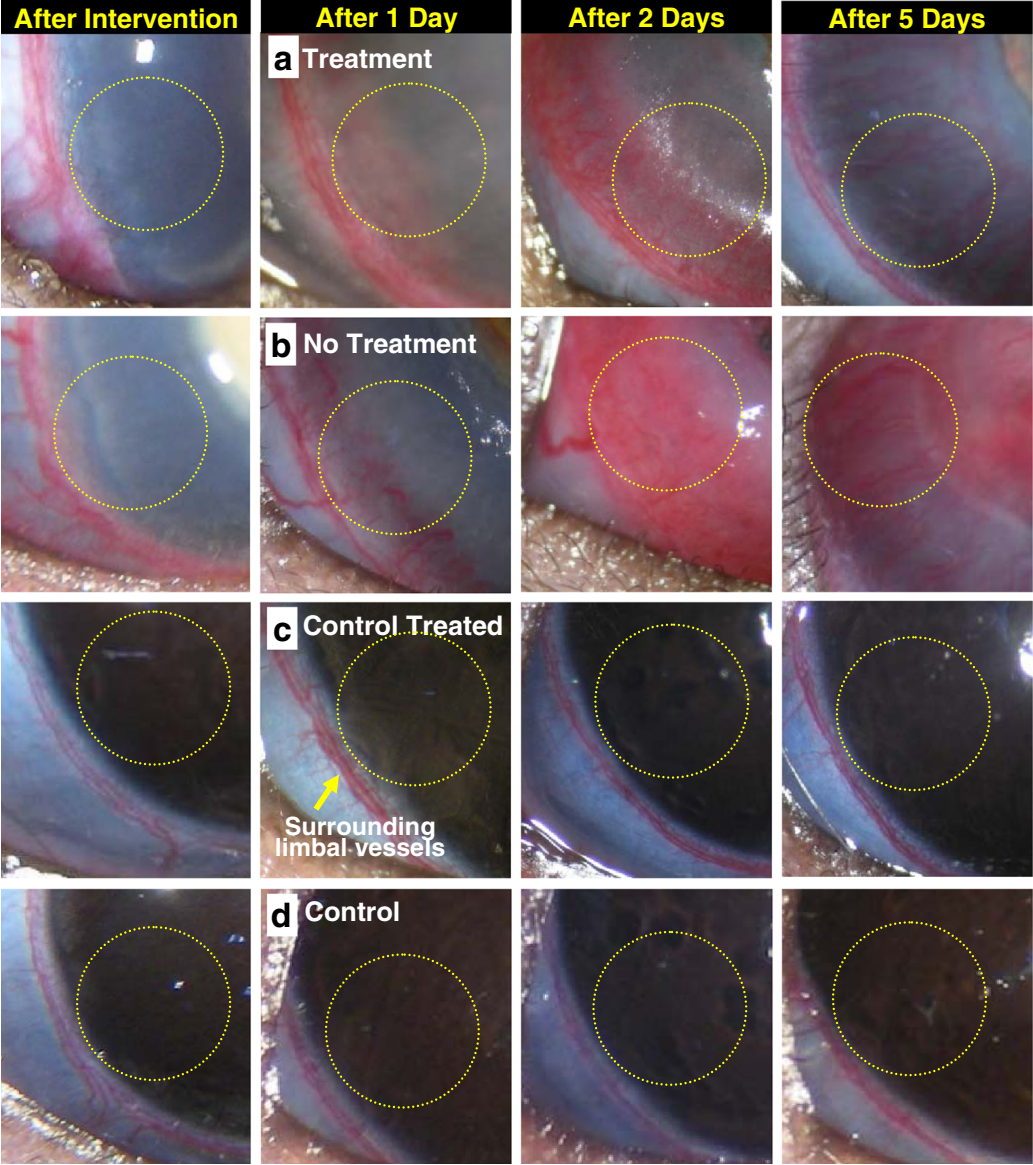
**a** Sequential optical images for the rat cornea intervened with silver nitrate cauterization before and after fs laser irradiation. For comparison, a positive control with neovascularization but without laser treatment, a negative control without neovascularization but with laser treatment, and a control without neovascularization and without laser treatment are also presented in (**b**), (**c**), and (**d**). *Yellow circles* indicate the region of the analysis for the percent reduction of neovascularization

A postoperative study to assess the effects of fs laser exposure on reduction of corneal neovascularization (CNV) has been conducted for rats with CNV induced by the silver nitrate cauterization model. Four rats were kept under observation for 5 days. For two rats that had corneas with CNV in both eyes, one eye was irradiated with fs laser pulses (Fig. 8a), while the other was kept as a positive control (*viz. induction of angiogenesis but no laser treatment*) (Fig. 8b). This might effectively remove the possible error of body response effects while estimating the fs laser-assisted % reduction of CNV as a function of following days of treatment. In a similar manner, for two control rats without angiogenesis, one eye was fs-irradiated (Fig. 8c) while the other was kept as a control (Fig. 8d).

For documentation of short-term postoperative changes, sequential optical images of rat cornea with and without neovascular structures were taken 1, 2, and 5 days after fs pulse laser treatment. In all animals, the vascular hemorrhages were resolved after 1 day and completely vanished over time. As a result, the % reduction in neovascularization was quantified after 1 day of treatment in order to avoid the pseudoeffect of hemorrhages in the Red % intensity values. From the injured area, no corneal infectious keratitis or inflammatory disorders were recorded in any of the examined rat corneas. No change in iris shape was observed. Moreover, the angiogenic response was uniform and almost identical to the early appearance of corneal injuries. Within 1 or 2 days, reduction in neovascular structures was observed in treated eyes compared to the positive control. No adverse effects of fs pulse irradiations were found in the control rat cornea under the current laser fluence (8.6 J/cm^2^), which is 2-fold higher than MVL threshold values for corneal stroma. It should be noted that the silver nitrate cauterization model recalls all the major steps followed in blood vessel formation in a reproducible manner and vessel formation continued for more than 7 days, at least [2], as mentioned in the experimental sections.

**Fig. 9:**
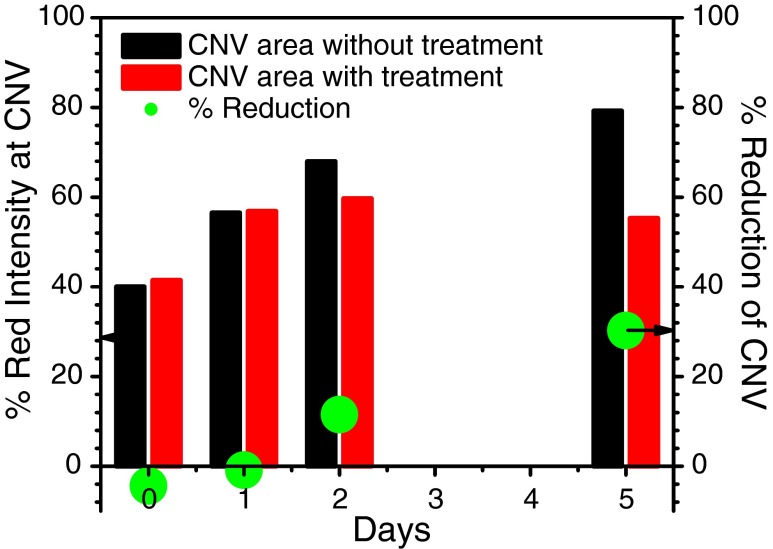
Plot of the red intensity of fs laser-treated cornea (*red-filled bar*) and untreated cornea (*black-filled bar*) and the percent reduction (*green-filled circles*) of neovascular structures as a function of elapsed time after fs laser irradiation with laser fluence of 8.6 J/cm^2^. CNVs were induced by silver nitrate cauterization. The value of the percent reduction was determined at the site (*yellow circles*) in the rat corneal images shown in Fig. 8

In Fig. 9, the reduction of CNV after laser microsurgery was determined by estimating the percent red intensity using Eq. (2) and measurement at the site of treatment from the rat corneal images shown in Fig. 8. After 2 days of treatment, about a 10 % reduction of CNV was observed at the site of fs laser exposure. The % reduction of CNV increased to 30 % by the 5th day of fs laser treatment without any assisted chemical agents. Holzer et al. [4] reported that, in the rabbits receiving verteporfin-assisted photodynamic therapy (vPDT) using cw-diode laser fluence of 17 or 50 J/cm^2^ resulted in 30 to 50 % short-term regression (3 to 5 days) of corneal neovascularization. In the case of human patients receiving vPDT, long-term regression of CNV was induced with application of the maximum effective laser fluence (150 J/cm^2^), which is three times higher than that used to treat choroidal neovascularization [46]. Baer and Foster reported good success in treating corneal vascularization with a 577-nm yellow dye laser (20.4 J/cm^2^, 50 μm spot size, and 0.05–0.2 s pulse duration) in patients with refractory graft rejection [47]. Under these laser irradiation operation conditions, possible complications of treatment include intrastromal hemorrhage, iris atrophy, and corneal thinning [47]. However, the fluence employed in our system (8.6 J/cm^2^) is lower than the existing fluences. One of the possible reasons for this is shortened pulse width (150 fs) [11, 12, 14–17, 22] along with the difference in spectral range utilized in our system (800 nm).

Brooks et al. (4.5 J/cm^2^ for 300 s) reported that the utilization of a 689-nm laser assisted with 3.65 mg/m^2^ verteporfin yielded apparent regression of corneal neovascularization [48]. However, the adverse reactions associated with verteporfin include visual disturbances, injection site reactions, and photosensitivity reactions. Further, photosensitization leads to absorption of laser energy by blood vessels at sites other than CNV, which may cause undesirable damage [11, 48]. In the current study, no such photosensitizers were utilized, and the results of reduction in neovascularizations are nonetheless comparable.

Since the model used in the current study reflects active angiogenesis, this could be treated with anti-vascular endothelial growth factor (anti-VEGF) medication. However, Ford et al. recently reported that inhibiting anti-VEGF might have a harmful effect on the tissues responsible for producing the fluid in the anterior segment of eye, namely, ciliary body and capillaries that have specialization called fenestration [49]. This may further lead to vision impairment. Based on these findings and considerations, fs laser pulses are a potentially viable method to treat these angiogenic structures without the use of any chemical agents. Furthermore, to extract the effects of fs laser treatment, a more precise stimulus to control the induction of neovascularizations is required [50].

## Conclusions

In the eye, corneal neovascularization diseases are associated with severe visual impairment and represent a major public health problem. Photocoagulation treatment for CNV has been reported, but this method has inadequate effects due to a high incidence of thermal damage to adjacent tissue. Femtosecond lasers (800 nm) are meanwhile a promising tool for minimally invasive intrastromal surgery by inducing plasma-mediated nonthermal tissue ablations. In this report, we introduce the concept of selectivity in the field of fs laser-assisted corneal microsurgery to treat neovascular structures intervened within the corneal intrastromal region of a rat eye, under in vivo conditions. The minimum visible laser lesion threshold over the neovascular region was determined as 4.3 J/cm^2^. Histological and OCT characterization of the anterior segment after fs laser exposure within the corneal stroma showed localized degeneration of CNV. No significant distortions were observed in other regions of the anterior segment such as the iris, corneal epithelium, corneal endothelium, etc. After laser exposure, rat corneas were monitored for 5 days to study the successive reduction of neovascular structures. After 2 days of treatment, about 10 % reduction of CNV was observed at the site of fs laser exposure. The degree of % reduction was increased to 30 % by the 5th day of fs laser treatment. It should be noted that successive in vivo studies are required to reinstate the findings of the current study (four to five raster scans of fs laser pulses (8.6 J/cm^2^)) for long-term reduction of corneal neovascularization within the corneal stroma.
